# Supplementary material for: Identification of potential insect ecological interactions using a metabarcoding approach
Source: PeerJ. 2025 Feb 17;13:e18906. doi: 10.7717/peerj.18906 (PMC11841592; doi:10.7717/peerj.18906)
Supplement: Supplemental Information 1 [file peerj-13-18906-s001.docx]

# Appendix A: Supplemental Tables

## Spongy Moth Tables

**Table S1. All taxa identified using the ITS target region across all spongy moth samples.** The frequency of taxa identified in spongy moth samples (9 each of egg and larva) across the egg and larva life stages using the ITS target region.

| Kingdom | Phylum | Class | Order | Family | Genus | Egg Frequency | Larva Frequency |
| --- | --- | --- | --- | --- | --- | --- | --- |
| Fungi | Ascomycota | Dothideomycetes | Pleosporales | Didymosphaeriaceae | *Paraconiothyrium* | 0 | 1 |
|  |  |  |  | Leptosphaeriaceae | *Leptosphaeria* | 1 | 0 |
|  |  |  |  | Lophiostomataceae | *Lophiostoma* | 1 | 0 |
|  |  |  |  |  | *(blank)* | 0 | 1 |
|  |  |  |  | Pleosporaceae | *Alternaria* | 6 | 6 |
|  |  |  |  |  | *Stemphylium* | 2 | 2 |
|  |  |  |  | (blank) | *(blank)* | 2 | 0 |
|  |  |  | (blank) | (blank) | *(blank)* | 2 | 2 |
|  |  | Eurotiomycetes | Onygenales | Ascosphaeraceae | *Ascosphaera* | 1 | 0 |
|  |  | Lecanoromycetes | Lecanorales | Parmeliaceae | *Punctelia* | 5 | 3 |
|  |  | Saccharomycetes | Saccharomycetales | Pichiaceae | *Pichia* | 2 | 0 |
|  |  | (blank) | (blank) | (blank) | *(blank)* | 6 | 3 |
|  | Basidiomycota | Agaricomycetes | Polyporales | Fomitopsidaceae | *Fomitopsis* | 0 | 1 |
|  |  | Microbotryomycetes | Sporidiobolales | Sporidiobolaceae | *Sporobolomyces* | 1 | 0 |
|  | (blank) | (blank) | (blank) | (blank) | *(blank)* | 8 | 5 |
| Plantae | Chlorophyta | Trebouxiophyceae | Trebouxiales | Trebouxiaceae | *Asterochloris* | 1 | 0 |
|  |  |  |  |  | *Symbiochloris* | 1 | 0 |
|  |  |  |  |  | *Trebouxia* | 6 | 2 |
|  |  |  | Unclassified | Unclassified | *Coccomyxa* | 1 | 0 |
|  | Tracheophyta | Liliopsida | Poales | Poaceae | *Bromus* | 1 | 0 |
|  |  |  |  |  | *Lolium* | 0 | 1 |
|  |  |  |  |  | *Poa* | 1 | 1 |
|  |  |  |  |  | *(blank)* | 0 | 1 |
|  |  | Magnoliopsida | Asterales | Asteraceae | *Centaurea* | 1 | 0 |
|  |  |  |  |  | *Coreopsis* | 1 | 1 |
|  |  |  |  |  | *Erigeron* | 1 | 0 |
|  |  |  |  |  | *Sonchus* | 1 | 1 |
|  |  |  |  |  | *(blank)* | 1 | 1 |
|  |  |  | Fabales | Fabaceae | *Lotus* | 1 | 2 |
|  |  |  |  |  | *Trifolium* | 2 | 1 |
|  |  |  |  |  | *Vicia* | 1 | 1 |
|  |  |  | Fagales | Betulaceae | *Betula* | 5 | 3 |
|  |  |  |  | Fagaceae | *Quercus* | 0 | 1 |
|  |  |  | Malpighiales | Salicaceae | *Populus* | 0 | 1 |
|  |  |  |  |  | *Salix* | 0 | 2 |
|  |  |  | Myrtales | Onagraceae | *Chamaenerion* | 0 | 1 |
|  |  |  |  |  | *Circaea* | 1 | 0 |
|  |  |  |  |  | *(blank)* | 0 | 1 |
|  |  |  | Ranunculales | Ranunculaceae | *Ranunculus* | 1 | 0 |
|  |  |  | Rosales | Cannabaceae | *Cannabis* | 1 | 0 |
|  |  |  |  | Rosaceae | *Potentilla* | 1 | 1 |
|  |  |  |  |  | *Prunus* | 0 | 1 |
|  |  |  |  |  | *Rosa* | 2 | 1 |
|  |  |  |  |  | *Sorbaria* | 0 | 1 |
|  |  |  | Saxifragales | Paeoniaceae | *Paeonia* | 2 | 1 |
|  |  |  | Solanales | Solanaceae | *Calibrachoa* | 1 | 1 |
|  |  | Pinopsida | Cupressales | Cupressaceae | *Juniperus* | 1 | 0 |
|  |  |  |  |  | *Thuja* | 1 | 1 |
|  | (blank) | (blank) | (blank) | (blank) | *(blank)* | 4 | 1 |

**Table S2. All taxa identified using the 16S target region across all spongy moth samples.** The frequency of taxa identified in spongy moth samples (9 each of egg and larva) across the egg and larva life stages using the 16S target region.

| Kingdom | Phylum | Class | Order | Family | Genus | Egg Frequency | Larva Frequency |
| --- | --- | --- | --- | --- | --- | --- | --- |
| Bacteria | Abditibacteriota | Abditibacteriia | Abditibacteriales | Abditibacteriaceae | Abditibacterium | 6 | 7 |
|  | Acidobacteriota | Terriglobia | Terriglobales | Acidobacteriaceae | Bryocella | 0 | 1 |
|  |  |  |  |  | Granulicella | 2 | 4 |
|  |  |  |  |  | Terriglobus | 0 | 1 |
|  | Actinomycetota | Acidimicrobiia | Acidimicrobiales | Iamiaceae | Aquihabitans | 1 | 0 |
|  |  | Actinomycetes | Propionibacteriales | Nocardioidaceae | Marmoricola | 1 | 1 |
|  |  |  | Jatrophihabitantales | Jatrophihabitantaceae | Jatrophihabitans | 0 | 3 |
|  |  |  | Kineosporiales | Kineosporiaceae | Kineosporia | 1 | 0 |
|  |  |  | Nakamurellales | Nakamurellaceae | Nakamurella | 1 | 1 |
|  |  |  | Streptosporangiales | Treboniaceae | Trebonia | 0 | 1 |
|  |  |  | Mycobacteriales | Nocardiaceae | Williamsia | 0 | 1 |
|  |  | Thermoleophilia | Solirubrobacterales | Patulibacteraceae | Patulibacter | 0 | 1 |
|  |  |  |  | Baekduiaceae | Baekduia | 1 | 1 |
|  | Armatimonadota | Armatimonadia | Capsulimonadales | Capsulimonadaceae | Capsulimonas | 4 | 2 |
|  |  | Fimbriimonadia | Fimbriimonadales | Fimbriimonadaceae | Fimbriimonas | 4 | 3 |
|  | Bacteroidota | Chitinophagia | Chitinophagales | Chitinophagaceae | Segetibacter | 1 | 0 |
|  |  | Cytophagia | Cytophagales | Cytophagaceae | Spirosoma | 2 | 2 |
|  |  | Sphingobacteriia | Sphingobacteriales | Sphingobacteriaceae | Pedobacter | 1 | 1 |
|  | Candidatus Saccharibacteria | Candidatus Saccharimonadia | Candidatus Saccharimonadales | Candidatus Saccharimonadaceae | Saccharimonas | 0 | 1 |
|  | Myxococcota | Polyangia | Polyangiales | Labilitrichaceae | Labilithrix | 0 | 1 |
|  | Pseudomonadota | Alphaproteobacteria | Hyphomicrobiales | Beijerinckiaceae | Methylovirgula | 0 | 1 |
|  |  |  |  | Lichenihabitantaceae | Lichenihabitans | 7 | 7 |
|  |  |  |  | Methylobacteriaceae | Enterovirga | 1 | 0 |
|  |  |  |  |  | Psychroglaciecola | 3 | 5 |
|  |  |  |  | Nitrobacteraceae | Tardiphaga | 1 | 2 |
|  |  |  |  | Aurantimonadaceae | Aureimonas | 1 | 1 |
|  |  |  |  | Phyllobacteriaceae | Mesorhizobium | 1 | 0 |
|  |  |  |  | Rhizobiaceae | Neorhizobium | 1 | 1 |
|  |  |  | Rhodospirillales | Acetobacteraceae | Roseomonas | 1 | 5 |
|  |  |  | Rickettsiales | Anaplasmataceae | Wolbachia | 4 | 1 |
|  |  |  | Sphingomonadales | Sphingomonadaceae | Sphingomicrobium | 1 | 0 |
|  |  |  | Geminicoccales | Geminicoccaceae | Geminicoccus | 1 | 0 |
|  |  | Betaproteobacteria | Burkholderiales | Oxalobacteraceae | Massilia | 5 | 3 |
|  |  |  |  | Burkholderiaceae | Caballeronia | 1 | 0 |
|  |  |  |  | Burkholderiales genera incertae sedis | Rhizobacter | 3 | 2 |
|  |  | Gammaproteobacteria | Oceanospirillales | Oceanospirillaceae | Marinomonas | 1 | 0 |
|  |  |  | Aeromonadales | Aeromonadaceae | Pseudaeromonas | 0 | 1 |
|  |  |  |  |  | Tolumonas | 0 | 2 |
|  | Bacillota | Clostridia | Eubacteriales | Oscillospiraceae | Ruminiclostridium | 1 | 0 |
|  | Gemmatimonadota | Gemmatimonadia | Gemmatimonadales | Gemmatimonadaceae | Gemmatirosa | 1 | 0 |
|  | Deinococcota | Deinococci | Trueperales | Trueperaceae | Truepera | 1 | 1 |

**Table S3. All taxa identified using the COI target region across all spongy moth samples.** The frequency of taxa identified in spongy moth samples (9 each of egg and larva) across the egg and larva life stages using the COI target region.

| Kingdom | Phylum | Class | Order | Family | Genus | Egg Frequency | Larva Frequency |
| --- | --- | --- | --- | --- | --- | --- | --- |
| Animalia | Arthropoda | Insecta | Blattodea | Blattellidae | *Blattella* | 0 | 1 |
|  |  |  | Coleoptera | Nitidulidae | *Epuraea* | 1 | 0 |
|  |  |  | Diptera | Cecidomyiidae | *Lestodiplosis* | 1 | 0 |
|  |  |  | Hemiptera | Adelgidae | *Pineus* | 1 | 0 |
|  |  |  |  | Miridae | *Lygus* | 4 | 2 |
|  |  |  | Lepidoptera | Erebidae | *(blank)* | 0 | 1 |
|  |  |  |  | Geometridae | *(blank)* | 1 | 0 |
|  |  |  | Neuroptera | Hemerobiidae | *Hemerobius* | 1 | 2 |

**Table S4. All taxa identified using the rbcL target region across all spongy moth samples.** The frequency of taxa identified in spongy moth samples (9 each of egg and larva) across the egg and larva life stages using the rbcL target region.

| Kingdom | Phylum | Class | Order | Family | Genus | Egg Frequency | Larva Frequency |
| --- | --- | --- | --- | --- | --- | --- | --- |
| Plantae | Chlorophyta | Trebouxiophyceae | Unclassified | Unclassified | *Coccomyxa* | 1 | 1 |
|  | Streptophyta | (blank) | (blank) | (blank) | *(blank)* | 4 | 1 |
|  | Tracheophyta | Liliopsida | Poales | Poaceae | *(blank)* | 1 | 1 |
|  |  |  | Zingiberales | Musaceae | *(blank)* | 1 | 0 |
|  |  | Magnoliopsida | Asterales | Asteraceae | *(blank)* | 3 | 2 |
|  |  |  | Caryophyllales | Caryophyllaceae | *(blank)* | 1 | 0 |
|  |  |  | Dipsacales | Adoxaceae | *Sambucus* | 1 | 2 |
|  |  |  | Fabales | Fabaceae | *Medicago* | 1 | 0 |
|  |  |  |  |  | *Securigera* | 1 | 0 |
|  |  |  |  |  | *Trifolium* | 1 | 0 |
|  |  |  |  |  | *(blank)* | 2 | 0 |
|  |  |  | Fagales | Betulaceae | *(blank)* | 5 | 5 |
|  |  |  |  | Fagaceae | *Fagus* | 2 | 2 |
|  |  |  |  |  | *(blank)* | 0 | 1 |
|  |  |  | Laurales | Lauraceae | *(blank)* | 0 | 2 |
|  |  |  | Malpighiales | Salicaceae | *Populus* | 3 | 4 |
|  |  |  |  |  | *Salix* | 2 | 0 |
|  |  |  | Rosales | Cannabaceae | *Cannabis* | 1 | 0 |
|  |  |  |  | Rosaceae | *Rosa* | 0 | 2 |
|  |  |  |  | Ulmaceae | *Ulmus* | 0 | 1 |
|  |  |  | Sapindales | Sapindaceae | *Acer* | 6 | 2 |
|  |  |  |  |  | *(blank)* | 0 | 1 |
|  |  |  | Vitales | Vitaceae | *(blank)* | 0 | 3 |
|  |  |  | (blank) | (blank) | *(blank)* | 2 | 2 |
|  |  | Pinopsida | Cupressales | Cupressaceae | *(blank)* | 2 | 0 |
|  |  |  | Pinales | Pinaceae | *Picea* | 5 | 5 |
|  |  |  |  |  | *Pinus* | 2 | 3 |

## Emerald Ash Borer Tables

**Table S5. All taxa identified using the ITS target region across all emerald ash borer samples.** The frequency of taxa identified in emerald ash borer samples across all life stages (3 sizes of larva, pupa, and adult) using the ITS target region.

| Kingdom | Phylum | Class | Order | Family | Genus | Larva1 Freq. | Larva2 Freq. | Larva3 Freq. | Pupa Freq. | Adult Freq. |
| --- | --- | --- | --- | --- | --- | --- | --- | --- | --- | --- |
| Fungi | Ascomycota | Candelariomycetes | Candelariales | Candelariaceae | Candelaria | 0 | 2 | 0 | 0 | 0 |
|  |  | Dothideomycetes | Botryosphaeriales | Botryosphaeriaceae | (blank) | 1 | 1 | 0 | 1 | 0 |
|  |  |  | Pleosporales | Cucurbitariaceae | Parafenestella | 0 | 1 | 0 | 0 | 0 |
|  |  |  |  |  | Pyrenochaeta | 1 | 4 | 0 | 0 | 1 |
|  |  |  |  | Pleosporaceae | Alternaria | 0 | 0 | 0 | 0 | 2 |
|  |  | Eurotiomycetes | Eurotiales | (blank) | (blank) | 0 | 0 | 0 | 0 | 1 |
|  |  | Lecanoromycetes | (blank) | (blank) | (blank) | 0 | 1 | 0 | 0 | 0 |
|  |  | Leotiomycetes | Helotiales | Dermateaceae | Neofabraea | 1 | 4 | 1 | 9 | 5 |
|  |  |  |  | Helotiales incertae sedis | Cadophora | 0 | 0 | 0 | 1 | 0 |
|  |  |  |  | (blank) | (blank) | 0 | 0 | 0 | 1 | 0 |
|  |  |  | Leotiales | Tympanidaceae | Tympanis | 0 | 0 | 0 | 0 | 1 |
|  |  | Orbiliomycetes | Orbiliales | Orbiliaceae | (blank) | 0 | 0 | 0 | 2 | 0 |
|  |  | Saccharomycetes | Saccharomycetales | Debaryomycetaceae | Yamadazyma | 0 | 0 | 0 | 0 | 2 |
|  |  | Sordariomycetes | Diaporthales | Diaporthaceae | Diaporthe | 0 | 2 | 0 | 1 | 0 |
|  |  |  |  | Valsaceae | Valsa | 2 | 4 | 3 | 7 | 2 |
|  |  |  |  | (blank) | (blank) | 0 | 1 | 0 | 0 | 0 |
|  |  |  | Hypocreales | Cordycipitaceae | Beauveria | 0 | 1 | 0 | 0 | 1 |
|  |  |  |  | Hypocreales incertae sedis | Eucasphaeria | 0 | 2 | 0 | 6 | 4 |
|  |  |  |  | Nectriaceae | Fusarium | 3 | 2 | 1 | 1 | 3 |
|  |  |  |  | Niessliaceae | Niesslia | 0 | 0 | 0 | 1 | 0 |
|  |  |  | Togniniales | Togniniaceae | Phaeoacremonium | 0 | 2 | 0 | 6 | 4 |
|  |  |  | (blank) | (blank) | (blank) | 0 | 0 | 1 | 0 | 0 |
|  |  | (blank) | (blank) | (blank) | (blank) | 3 | 3 | 0 | 2 | 4 |

**Table S6. All taxa identified using the 16S target region across all emerald ash borer samples.** The frequency of taxa identified in emerald ash borer samples across all life stages (3 sizes of larva, pupa, and adult) using the 16S target region.

| Kingdom | Phylum | Class | Order | Family | Genus | Larva1 Freq. | Larva2 Freq, | Larva3 Freq, | Pupa Freq, | Adult Freq, |
| --- | --- | --- | --- | --- | --- | --- | --- | --- | --- | --- |
| Bacteria | Acidobacteriota | Terriglobia | Terriglobales | Acidobacteriaceae | Terriglobus | 0 | 0 | 0 | 0 | 3 |
|  |  |  |  |  | Edaphobacter | 0 | 0 | 0 | 0 | 1 |
|  |  |  |  |  | Granulicella | 0 | 0 | 0 | 0 | 1 |
|  | Bacteroidota | Chitinophagia | Chitinophagales | Chitinophagaceae | Ginsengibacter | 0 | 0 | 0 | 1 | 0 |
|  |  | Flavobacteriia | Flavobacteriales | Crocinitomicaceae | Taishania | 0 | 0 | 0 | 2 | 0 |
|  |  | Cytophagia | Cytophagales | Fulvivirgaceae | Chryseolinea | 0 | 0 | 0 | 1 | 1 |
|  |  | Sphingobacteriia | Sphingobacteriales | Sphingomonadaceae | Sphingobium | 1 | 0 | 0 | 0 | 0 |
|  |  |  |  | Sphingobacteriaceae | Olivibacter | 4 | 3 | 1 | 4 | 6 |
|  |  |  |  |  | Pedobacter | 1 | 0 | 0 | 0 | 3 |
|  | Bdellovibrionota | Oligoflexia | Oligoflexales | Oligoflexaceae | Oligoflexus | 0 | 0 | 0 | 1 | 0 |
|  | Myxococcota | Polyangia | Polyangiales | Labilitrichaceae | Labilithrix | 0 | 0 | 0 | 0 | 1 |
|  | Pseudomonadota | Alphaproteobacteria | Hyphomicrobiales | Beijerinckiaceae | Methylovirgula | 0 | 0 | 0 | 0 | 2 |
|  |  |  |  | Nitrobacteraceae | Tardiphaga | 1 | 1 | 0 | 1 | 4 |
|  |  |  |  | Rhizobiaceae | Neorhizobium | 7 | 17 | 1 | 18 | 19 |
|  |  |  |  | Aurantimonadaceae | Aureimonas | 0 | 0 | 0 | 0 | 0 |
|  |  |  |  | Methylopilaceae | Hansschlegelia | 0 | 2 | 1 | 1 | 1 |
|  |  |  | Sphingomonadales | Sphingomonadaceae | Allosphingosinicella | 0 | 0 | 0 | 1 | 0 |
|  |  |  |  |  | Novosphingobium | 1 | 0 | 0 | 0 | 1 |
|  |  |  | Rickettsiales | Anaplasmataceae | Wolbachia | 0 | 0 | 0 | 1 | 0 |
|  |  | Betaproteobacteria | Burkholderiales | Oxalobacteraceae | Duganella | 0 | 1 | 0 | 0 | 1 |
|  |  | Gammaproteobacteria | Enterobacterales | Pectobacteriaceae | Acerihabitans | 1 | 0 | 0 | 0 | 3 |
|  |  |  | Lysobacterales | Rhodanobacteraceae | Dokdonella | 1 | 0 | 0 | 0 | 1 |

**Table S7. All taxa identified using the COI target region across all emerald ash borer samples.** The frequency of taxa identified in emerald ash borer samples across all life stages (3 sizes of larva, pupa, and adult) using the COI target region.

| Kingdom | Phylum | Class | Order | Family | Larva1 Freq. | Larva2 Freq. | Larva3 Freq. | Pupa Freq. | Adult Freq. |
| --- | --- | --- | --- | --- | --- | --- | --- | --- | --- |
| Animalia | Arthropoda | Arachnida | Sarcoptiformes | Oppiidae | 0 | 0 | 0 | 0 | 1 |
|  |  |  | Trombidiformes | Eupodidae | 0 | 0 | 0 | 0 | 1 |
|  |  | Insecta | Diptera | Cecidomyiidae | 0 | 1 | 0 | 0 | 0 |
|  |  |  | Hymenoptera | Ichneumonidae | 0 | 1 | 1 | 1 | 0 |

**Table S8. All taxa identified using the 18S target region across all emerald ash borer samples.** The frequency of taxa identified in emerald ash borer samples across all life stages (3 sizes of larva, pupa, and adult) using the 18S target region.

| Kingdom | Phylum | Class | Order | Family | Genus | Larva1 Freq. | Larva2 Freq. | Larva3 Freq. | Pupa Freq. | Adult Freq. |
| --- | --- | --- | --- | --- | --- | --- | --- | --- | --- | --- |
| Animalia | Arthropoda | Insecta | Hymenoptera | (blank) | (blank) | 2 | 1 | 0 | 4 | 0 |
|  | Nematoda | Chromadorea | Rhabditida | Diplogasteridae | Rhabditolaimus | 1 | 1 | 0 | 0 | 0 |
|  |  |  |  | Neotylenchidae | (blank) | 0 | 3 | 1 | 0 | 1 |
|  |  |  |  | Panagrolaimidae | Panagrellus | 0 | 1 | 0 | 0 | 0 |
|  |  |  |  | (blank) | (blank) | 0 | 0 | 0 | 0 | 1 |
| Fungi | Ascomycota | Dothideomycetes | Botryosphaeriales | Botryosphaeriaceae | (blank) | 0 | 0 | 0 | 1 | 0 |
|  |  |  | Pleosporales | (blank) | (blank) | 0 | 1 | 0 | 0 | 0 |
|  |  | Leotiomycetes | Helotiales | (blank) | (blank) | 0 | 1 | 0 | 1 | 0 |
|  |  |  | (blank) | (blank) | (blank) | 1 | 0 | 0 | 0 | 0 |
|  |  | Saccharomycetes | Saccharomycetales | (blank) | (blank) | 0 | 0 | 0 | 0 | 1 |
|  |  | Sordariomycetes | Diaporthales | Valsaceae | Diaporthe | 0 | 2 | 0 | 0 | 0 |
|  |  |  | Hypocreales | Cordycipitaceae | (blank) | 0 | 0 | 0 | 0 | 1 |
|  |  |  |  | Nectriaceae | (blank) | 1 | 0 | 0 | 0 | 0 |
|  |  |  |  | Niessliaceae | Niesslia | 0 | 1 | 0 | 0 | 1 |
|  |  |  |  | (blank) | (blank) | 0 | 1 | 0 | 0 | 0 |
|  |  | (blank) | (blank) | (blank) | (blank) | 1 | 0 | 0 | 0 | 0 |
| Unknown | Apicomplexa | Conoidasida | Cryptosporida | Cryptosporidiidae | Cryptosporidium | 0 | 0 | 0 | 1 | 0 |

Native Pollinator Larva Tables

**Table S9. All taxa identified using the rbcL region across larval samples taken from bee homes.**

| Phylum | Class | Order | Family | Genus |
| --- | --- | --- | --- | --- |
| Tracheophyta | Liliopsida | Asparagales | Iridaceae | *Iris* |
|  |  |  | Orchidaceae | *Cypripedium* |
|  | Magnoliopsida | Apiales | Apiaceae | *Daucus* |
|  |  | Asterales | Asteraceae | *Arctium* |
|  |  |  |  | *Crepis* |
|  |  |  |  | *Erigeron* |
|  |  |  |  | *Helianthus* |
|  |  |  |  | *Hypochaeris* |
|  |  |  |  | *Lactuca* |
|  |  |  |  | *Leucanthemum* |
|  |  |  |  | *Tanacetum* |
|  |  |  | Campanulaceae | *Campanula* |
|  |  | Brassicales | Brassicaceae | *Brassica* |
|  |  | Caryophyllales | Polygonaceae | *Persicaria* |
|  |  | Cornales | Cornaceae | *Cornus* |
|  |  |  | Hydrangeaceae | *Hydrangea* |
|  |  | Cucurbitales | Cucurbitaceae | *Cucumis* |
|  |  | Dipsacales | Caprifoliaceae | *Lonicera* |
|  |  |  |  | *Symphoricarpos* |
|  |  | Fabales | Fabaceae | *Glycine* |
|  |  |  |  | *Lathyrus* |
|  |  |  |  | *Lotus* |
|  |  |  |  | *Lupinus* |
|  |  |  |  | *Medicago* |
|  |  |  |  | *Melilotus* |
|  |  |  |  | *Pisum* |
|  |  |  |  | *Trifolium* |
|  |  | Lamiales | Oleaceae | *Fraxinus* |
|  |  |  | Plantaginaceae | *Veronica* |
|  |  | Malpighiales | Hypericaceae | *Hypericum* |
|  |  |  | Salicaceae | *Populus* |
|  |  |  |  | *Salix* |
|  |  |  | Violaceae | *Viola* |
|  |  | Malvales | Malvaceae | *Malva* |
|  |  | Myrtales | Onagraceae | *Circaea* |
|  |  |  |  | *Epilobium* |
|  |  |  |  | *Oenothera* |
|  |  | Oxalidales | Oxalidaceae | *Oxalis* |
|  |  | Ranunculales | Papaveraceae | *Papaver* |
|  |  |  | Ranunculaceae | *Anemone* |
|  |  |  |  | *Ranunculus* |
|  |  | Rosales | Rhamnaceae | *Frangula* |
|  |  |  | Rosaceae | *Fragaria* |
|  |  |  |  | *Potentilla* |
|  |  |  |  | *Prunus* |
|  |  |  |  | *Rosa* |
|  |  |  |  | *Rubus* |
|  |  |  |  | *Spiraea* |
|  |  | Sapindales | Sapindaceae | *Acer* |
|  |  | Saxifragales | Paeoniaceae | *Paeonia* |
|  |  | Solanales | Convolvulaceae | *Convolvulus* |
|  |  |  | Solanaceae | *Solanum* |
|  |  |  |  | *Withania* |
|  |  | Vitales | Vitaceae | *Vitis* |
|  |  | Zingiberales | Musaceae | *Musa* |
|  | Pinopsida | Pinales | Pinaceae | *Picea* |
|  |  |  |  | *Pinus* |

**Table S10. All plant taxa identified using the ITS target region amplified from extracts from lava in bee homes.**

| Phylum | Class | Order | Family | Genus |
| --- | --- | --- | --- | --- |
| Chlorophyta | Chlorophyceae | Chlamydomonadales | Chlamydomonadaceae | *Chlamydomonas* |
|  |  |  | Coccomyxaceae | *Coccomyxa* |
|  | Trebouxiophyceae | Trebouxiales | Trebouxiaceae | *Trebouxia* |
| Tracheophyta | Liliopside | Poales | Poaceae | *Bromus* |
|  | Magnoliopsida | Apiales | Apiaceae | *Anethum* |
|  |  |  |  | *Coriandrum* |
|  |  |  |  | *Seseli* |
|  |  | Asparagales | Amaryllidaceae | *Allium* |
|  |  | Asterales | Asteraceae | *Achillea* |
|  |  |  |  | *Anaphalis* |
|  |  |  |  | *Arctium* |
|  |  |  |  | *Artemisia* |
|  |  |  |  | *Bellis* |
|  |  |  |  | *Calendula* |
|  |  |  |  | *Centaurea* |
|  |  |  |  | *Cichorium* |
|  |  |  |  | *Cirsium* |
|  |  |  |  | *Coreopsis* |
|  |  |  |  | *Cosmos* |
|  |  |  |  | *Crepis* |
|  |  |  |  | *Dahlia* |
|  |  |  |  | *Doronicum* |
|  |  |  |  | *Echinacea* |
|  |  |  |  | *Echinops* |
|  |  |  |  | *Erigeron* |
|  |  |  |  | *Eupatorium* |
|  |  |  |  | *Euthamia* |
|  |  |  |  | *Heliopsis* |
|  |  |  |  | *Hieracium* |
|  |  |  |  | *Hypochaeris* |
|  |  |  |  | *Leontodon* |
|  |  |  |  | *Leucanthemum* |
|  |  |  |  | *Matricaria* |
|  |  |  |  | *Packera* |
|  |  |  |  | *Pilosella* |
|  |  |  |  | *Rudbeckia* |
|  |  |  |  | *Salidago* |
|  |  |  |  | *Silphium* |
|  |  |  |  | *Solidago* |
|  |  |  |  | *Sonchus* |
|  |  |  |  | *Symphyotrichum* |
|  |  |  |  | *Tanacetum* |
|  |  |  |  | *Taraxacum* |
|  |  |  |  | *Tripleurospermum* |
|  |  |  | Campanulaceae | *Campanula* |
|  |  | Boraginales | Boraginaceae | *Borago* |
|  |  |  |  | *Cynoglossum* |
|  |  |  |  | *Echium* |
|  |  |  |  | *Glandora* |
|  |  |  |  | *Lithospermum* |
|  |  |  |  | *Mertensia* |
|  |  |  |  | *Symphytum* |
|  |  | Brassicales | Brassicaceae | *Alliaria* |
|  |  |  |  | *Arabidopsis* |
|  |  |  |  | *Barbarea* |
|  |  |  |  | *Berteroa* |
|  |  |  |  | *Brassica* |
|  |  |  |  | *Capsella* |
|  |  |  |  | *Eruca* |
|  |  |  |  | *Erysimum* |
|  |  |  |  | *Hesperis* |
|  |  |  |  | *Iberis* |
|  |  |  |  | *Lepidium* |
|  |  |  |  | *Lobularia* |
|  |  |  |  | *Lunaria* |
|  |  |  |  | *Sisymbrium* |
|  |  |  |  | *Thelypodium* |
|  |  |  |  | *Thlaspi* |
|  |  |  | Tropaeolaceae | *Tropaeolum* |
|  |  | Caryophyllales | Amaranthaceae | *Celosia* |
|  |  |  |  | *Chenopodium* |
|  |  |  | Caryophyllaceae | *Cerastium* |
|  |  |  |  | *Dianthus* |
|  |  |  |  | *Petrorhagia* |
|  |  |  |  | *Silene* |
|  |  |  |  | *Stellaria* |
|  |  |  | Portulacaceae | *Portulaca* |
|  |  | Dipsacales | Adoxaceae | *Sambucus* |
|  |  |  |  | *Viburnum* |
|  |  | Ericales | Balsaminaceae | *Impatiens* |
|  |  |  | Clethraceae | *Clethra* |
|  |  |  | Ericaceae | *Erica* |
|  |  |  | Polemoniaceae | *Phlox* |
|  |  |  | Primulaceae | *Lysimachia* |
|  |  | Fabales | Fabaceae | *Anthyllis* |
|  |  |  |  | *Astragalus* |
|  |  |  |  | *Cytisus* |
|  |  |  |  | *Glycine* |
|  |  |  |  | *Hedysarum* |
|  |  |  |  | *Laburnum* |
|  |  |  |  | *Lathyrus* |
|  |  |  |  | *Lotus* |
|  |  |  |  | *Lupinus* |
|  |  |  |  | *Medicago* |
|  |  |  |  | *Melilotus* |
|  |  |  |  | *Securigera* |
|  |  |  |  | *Strophostyles* |
|  |  |  |  | *Trifolium* |
|  |  |  |  | *Vicia* |
|  |  |  | Betulaceae | *Betula* |
|  |  |  | Fagaceae | *Fagus* |
|  |  |  |  | *Quercus* |
|  |  | Lamiales | Lamiaceae | *Origanum* |
|  |  |  | Oleaceae | *Forsythia* |
|  |  |  |  | *Fraxinus* |
|  |  |  |  | *Ligustrum* |
|  |  |  |  | *Syringa* |
|  |  |  | Plantaginaceae | *Chaenorhinum* |
|  |  |  |  | *Linaria* |
|  |  |  |  | *Plantago* |
|  |  |  |  | *Veronica* |
|  |  |  |  | *Veronicastrum* |
|  |  |  | Scrophulariceae | *Sutera* |
|  |  | Malpighiales | Salicaceae | *Populus* |
|  |  |  |  | *Salix* |
|  |  |  | Violaceae | *Viola* |
|  |  | Malvales | Malvaceae | *Abutilon* |
|  |  |  |  | *Malva* |
|  |  | Myrtales | Lythraceae | *Lythrum* |
|  |  |  | Onagraceae | *Chamaenerion* |
|  |  |  |  | *Circaea* |
|  |  |  |  | *Epilobium* |
|  |  |  |  | *Oenothera* |
|  |  | Poales | Cyperaceae | *Carex* |
|  |  |  | Poaceae | *Aegilops* |
|  |  |  |  | *Agrostis* |
|  |  |  |  | *Festuca* |
|  |  |  |  | *Lolium* |
|  |  |  |  | *Phalaris* |
|  |  |  |  | *Poa* |
|  |  |  |  | *Setaria* |
|  |  |  |  | *Triticum* |
|  |  | Ranunculales | Berberidaceae | *Berberis* |
|  |  |  | Papaveraceae | *Chelidonium* |
|  |  |  |  | *Lamprocapnos* |
|  |  |  | Ranunculaceae | *Actaea* |
|  |  |  |  | *Anemone* |
|  |  |  |  | *Clematis* |
|  |  |  |  | *Ranunculus* |
|  |  | Rosales | Cannabaceae | *Cannabis* |
|  |  |  | Rhamnaceae | *Frangula* |
|  |  |  | Rosaceae | *Dasiphora* |
|  |  |  |  | *Filipendula* |
|  |  |  |  | *Fragaria* |
|  |  |  |  | *Geum* |
|  |  |  |  | *Malus* |
|  |  |  |  | *Potentilla* |
|  |  |  |  | *Prunus* |
|  |  |  |  | *Rosa* |
|  |  |  |  | *Rubus* |
|  |  |  |  | *Sorbaria* |
|  |  |  |  | *Sorbus* |
|  |  | Sapindales | Anacardiaceae | *Rhus* |
|  |  |  | Rutaceae | *Choisya* |
|  |  | Saxifragales | Crassulaceae | *Hylotelephium* |
|  |  |  |  | *Phedimus* |
|  |  |  |  | *Sedum* |
|  |  |  |  | *Sempervivum* |
|  |  |  | Paeoniaceae | *Paeonia* |
|  |  | Solanales | Convolvulaceae | *Convolvulus* |
|  |  |  | Solanaceae | *Calibrachoa* |
|  |  |  |  | *Petunia* |
|  |  | Vitales | Vitaceae | *Vitis* |
|  | Pinopsida | Pinales | Cupressaceae | *Callitropsis* |

**Table S11. All fungal taxa identified using the ITS target region amplified from extracts from lava in bee homes.**

| Phylum | Class | Order | Family | Genus |
| --- | --- | --- | --- | --- |
| Ascomycota | Dothideomycetes | Botryosphaeriales | Botryosphaeriaceae | *Diplodia* |
|  |  |  |  | *Fusarium* |
|  |  | Pleosporales | Phaeosphaeriaceae | *Phaeosphaeria* |
|  |  |  | Pleosporaceae | *Alternaria* |
|  |  |  |  | *Curvularia* |
|  |  |  |  | *Epicoccum* |
|  | Eurotiomycetes | Ascosphaerales | Ascosphaeraceae | *Ascosphaera* |
|  |  | Coryneliales | Eremascaceae | *Eremascus* |
|  |  | Eurotiales | Aspergillaceae | *Aspergillus* |
|  |  |  |  | *Penicillium* |
|  | Pezizomycetes | Pezizales | Pyronemataceae | *Heydenia* |
|  | Saccharomycetes | Saccharomycetales | Saccharomycetaceae | *Pichia* |
|  |  |  |  | *Starmerella* |
|  | Sordariomycetes | Microascales | Microascaceae | *Scopulariopsis* |
|  |  | Sordariales | Chaetomiaceae | *Trichocladium* |
|  |  | Trichosphaeriales | Trichosphaeriaceae | *Nigrospora* |
| Basidiomycota | Moniliellomycetes | Moniliellales | Moniliellaceae | *Moniliella* |
|  | Ustilaginomycetes | Ustilaginales | Ustilaginaceae | *Sporobolomyces* |
| Chlorophyta | Chlorophyceae | Chlamydomonadales | Chlamydomonadaceae | *Chlamydomonas* |
|  |  |  | Coccomyxaceae | *Coccomyxa* |
|  | Trebouxiophyceae | Trebouxiales | Trebouxiaceae | *Trebouxia* |
| Mucoromycota | Mucoromycetes | Mucorales | Mucoraceae | *Mucor* |

**Table S12. All taxa identified using the 16s target region amplified from extracts from lava in bee homes.**

| Phylum | Class | Order | Family | Genus |
| --- | --- | --- | --- | --- |
| Actinobacteriota | Actinomycetia | Actinomycetales | Bifidobacteriaceae | *Bifidobacterium* |
|  |  |  | Brevibacteriaceae | *Brevibacterium* |
|  |  |  | Micrococcaceae | *Arthrobacter* |
|  |  | Mycobacteriales | Pseudonocardiaceae | *Actinomycetospora* |
|  |  | Propionibacteriales | Nocardioidaceae | *Nocardioides* |
|  |  | Propionibacteriales | Propionibacteriaceae | *Cutibacterium* |
|  |  | Streptomycetales | Streptomycetaceae | *Streptomyces* |
|  | Coriobacteriia | Coriobacteriales | Coriobacteriaceae | *Collinsella* |
|  |  |  | Eggerthellaceae | *Slackia* |
| Bacteroidota | Bacteroidia | Bacteroidales | Marinilabiliaceae | *JC017* |
|  |  | Flavobacteriales | Weeksellaceae | *Kaistella* |
| Firmicutes | Bacilli | Bacillales | Amphibacillaceae | *Oceanobacillus* |
|  |  |  | Bacillaceae | *Bacillus* |
|  |  |  |  | *Priestia* |
|  |  |  | Planococcaceae | *Sporosarcina* |
|  |  | Lactobacillales | Enterococcaceae | *Melissococcus* |
|  |  |  | Lactobacillaceae | *Apilactobacillus* |
|  |  |  |  | *Fructobacillus* |
|  |  |  |  | *Holzapfelia* |
|  |  |  | Streptococcaceae | *Lactococcus* |
|  |  | Staphylococcales | Staphylococcaceae | *Staphylococcus* |
| Proteobacteria | Alphaproteobacteria | Acetobacterales | Acetobacteraceae | *Roseomonas* |
|  |  | Acetobacterales |  | *Saccharibacter* |
|  |  | Rhizobiales | Beijerinckiaceae | *Methylobacterium* |
|  |  | Rickettsiales | Anaplasmataceae | *Wolbachia* |
|  |  |  | Arcanobacteraceae | *Ac37b* |
|  |  |  | Rickettsiaceae | *Rickettsia* |
|  |  | Sphingomonadales | Sphingomonadaceae | *Sphingomicrobium* |
|  | Gammaproteobacteria | Burkholderiales | Burkholderiaceae | *Massilia* |
|  |  | Cardiobacteriales | Wohlfahrtiimonadaceae | *Ignatzschineria* |
|  |  | Enterobacterales | Enterobacteriaceae | *Arsenophonus* |
|  |  |  |  | *Buchnera* |
|  |  |  |  | *Erwinia* |
|  |  |  |  | *Gilliamella* |
|  |  |  |  | *Hamiltonella* |
|  |  |  |  | *Pantoea* |
|  |  |  |  | *Rosenbergiella* |
|  |  |  |  | *Sodalis* |
|  |  | Pseudomonadales | Moraxellaceae | *Acinetobacter* |
